# 1433. Impact of 2019 US Food and Drug Administration (FDA) Guidance on Developing Drugs for Urinary Tract Infection (UTI) on the Perceived Efficacy of Antibiotics for the Treatment of Uncomplicated UTI (uUTI)

**DOI:** 10.1093/ofid/ofab466.1625

**Published:** 2021-12-04

**Authors:** Anne Santerre Henriksen, Lindsay Nicolle, Anita F Das

**Affiliations:** 1 Maxel Consulting ApS, London, England, United Kingdom; 2 University of Manitoba, Winnipeg, Manitoba, Canada; 3 Adagio Therapeutics, Inc., Waltham, Massachusetts

## Abstract

**Background:**

In 2019, the FDA issued guidance on drug development for treatment of UTIs. To explore the impact of this guidance, we compared clinical and microbiological outcomes of the fluoroquinolones norfloxacin and ciprofloxacin and the β-lactams pivmecillinam and sulopenem for treatment of uUTIs from original publications versus recent analyses conducted in accordance with the FDA guidance.

**Methods:**

The efficacy of pivmecillinam 400 mg twice daily (BID), 3 days (3d) versus norfloxacin 400 mg BID, 3d was reported in a 2002 publication. Patient-level data were used to re-analyze clinical and microbiological outcomes in the microbiological intent-to-treat population in accordance with the 2019 FDA guidance. For descriptive comparison, we present the efficacy of ciprofloxacin 250 mg BID, 3d vs sulopenem 500 mg BID, 5d in the 2020 SURE-1 trial (also conducted in accordance with FDA guidance) alongside historical efficacy data for ciprofloxacin.

**Results:**

Re-analysis of data from the trial of pivmecillinam and norfloxacin showed microbiological responses for pivmecillinam and norfloxacin of 64% and 79%, respectively. Microbiological responses were higher, 75% for pivmecillinam and 91% for norfloxacin, in the original analysis. For clinical response, re-analysis showed 75% for pivmecillinam and 88% for norfloxacin, while historical data were 82% and 88%, respectively. In the SURE-1 trial, the microbiological response of patients assessed at Day 12 was 76.6% for sulopenem and 79.1% for ciprofloxacin. In a 2002 publication, bacterial eradication at 4 to 11 days after treatment was 93.7% for ciprofloxacin 250 mg, a higher response rate than that reported in SURE-1. For clinical response, rates were 78.7% for ciprofloxacin in SURE-1 and 92.7% for the historical ciprofloxacin data.

**Conclusion:**

When assessed in accordance with the 2019 FDA guidance, clinical and microbiological efficacy of both fluoroquinolones and β-lactam antibiotics appears lower than has been published in the past. Healthcare providers should be aware that newer antibiotics may appear to have a lower efficacy than older antibiotics due to the application of more stringent definitions in the FDA guidance.

**Disclosures:**

**Anne Santerre Henriksen, MS**, **Advanz** (Consultant)**Shionogi BV** (Consultant)**UTILITY Therapeutics** (Consultant) **Lindsay Nicolle, MD**, **Entos** (Consultant)**GSK** (Consultant)**Iterum** (Consultant)**Utility Therapeutics** (Consultant) **Anita F. Das, PhD**, **Adagio Therapeutics, Inc.** (Consultant)

